# The World after COVID-19: Reflections on Global Health and Policy

**DOI:** 10.5334/aogh.2902

**Published:** 2021-07-23

**Authors:** Nasser Yassin, Shadi Saleh

**Affiliations:** 1 Chairperson of the Health Management and Policy Department at the American University of Beirut, Lebanon; 2 Founding Director of the Global Health Institute at the American University of Beirut, Lebanon

## Abstract

COVID-19 has infected hundreds of millions of people across the globe. The pandemic has also inflicted serious damages on global and regional governing political structures to a degree meriting a revisit of their own raison d’etre. The global economic fallout is also unprecedented as the flows of goods and people got severely disrupted while lockdowns hit the transport, services and retail industries, among others. We argue that three realities need to be genuinely addressed for building a post COVID-19 order that has to be amply equipped to deal with the next global crisis, as well as the ones on-going for decades. First, there is need to shelf-away the hitherto practiced doctrine that global crises and problems are confronted through local responses. Second, the COVID-19 pandemic has cautioned us on the need to (re)invest in basic, many may consider naïve and simple, public health functions such as sanitation as well as transparent national and global health monitoring. Third, the pandemic is a clear reprimand to discard the mantra that privatization of healthcare delivery system is the solution in favor of viewing health as a public good that needs to be managed and executed by the state and its public sector, be it national, sub-regional or local. It is critical that we learn from such pandemic and advance our societies to become stronger.

As we write this commentary confined once again to our homes, the newsfeed keeps coming: COVID-19 has infected more than 102 million people, and still counting, across the globe [1]. The pandemic has also inflicted serious damages on global – and regional – governing political structures to a degree, meriting a revisit of their own raison d’etre and mode of operation (or lack thereof). The global economic fallout will also be unprecedented as the flows of goods and people were severely disrupted while lockdowns hit the transport, services, and retail industries, among others. In recent years, many around the world have hypothesized on the impact of surging nationalism globally on shaping a new world order [2]. COVID-19 revealed how nationalism can translate into breaking decade-long alliances presumably built on common values and interests. The question going forward is whether the world post-COVID-19 will support and continue with that nationalism theme or tilt the balance towards placing more value on globalist approaches and values, starting with better global health.

We argue that three stark realities need to be genuinely addressed for building a post-COVID-19 order that has to be amply equipped to deal with the next global crisis, as well as the ones that have been ongoing for decades. We are sure that other scholars will focus this discussion on many other areas, including global trade, philosophy of the nation-state, and the like. For the sake of this commentary, we will be focusing on three targeted areas relating to global health and its intersection with overall public policy.

First, there is need to shelve the hitherto practiced doctrine that global crises and problems are confronted through local responses. Such an approach has been the norm in the past decades, from the ways the world responded to refugee crises such as the Syrian crisis, or through the maneuvering we have seen around combating climate change during and after the Paris Agreement in 2016. So far, tackling the COVID-19 pandemic has been predominantly “Westphalian,” with a vivid comeback of the nation-state coupled with an undertone of surging nationalism. While it is understandable that states lead efforts to mitigate the pandemic and slow it down, battling it must happen at the global level, with multilateral collaboration and combined work.

Second, the COVID-19 pandemic has cautioned us on the need to (re)invest in basic, many may consider naïve and simple, public health functions such as sanitation, as well as transparent national and global health monitoring. The last decade has witnessed an overemphasis on technology as the savior and cure of all our ills, which is clearly not enough. We acknowledge the positive and tailored impact of medical technology advancement on many segments of the population. However, from a global cost-effectiveness lens, COVID-19 has proven it is more sensible and largely efficient to invest in the basics first. This can be considered a call to go back to the basics of public health across populations and communities.

Third, the pandemic is a clear reprimand to discard the mantra that privatization of healthcare delivery system is the solution in favor of viewing health as a public good that needs to be managed and executed by the state and its public sector, be it national, sub-regional, or local. The COVID-19 pandemic has clearly proven the cruciality of the “health for all” approach, a perspective that views health as public good. Thus, the expectation of the population of every single country hit with COVID-19 was that the state should take care of them, from testing, containment, and provision of health care, to bringing citizens back to their country. This was even true for countries whose prevailing culture and philosophy valued a minimalist state intervention in all aspects of life. COVID-19 will change how all of us view the role and responsibility of the state. Hopefully, moving forward will result in many of us working towards having a better, more efficient and effective public sector that prioritizes science and evidence over short-term political gain and futile global positioning and competition.

COVID-19 is neither the first global crisis that humanity has witnessed, nor it will be its last. The critical issue at the moment is to learn from this pandemic and advance our societies to become stronger and more just, something that the world has not been prioritizing in the past decades. This is, perhaps, a wake-up call.

## References

[1] Johns Hopkins University Coronavirus Resource Center. COVID-19 Dashboard by the Center for Systems Science and Engineering (CSSE) at Johns Hopkins University (JHU). https://coronavirus.jhu.edu/map.html. Accessed on January 31, 2021.

[2] Foreign Policy. How the World Will Look After the Coronavirus Pandemic 2020. https://foreignpolicy.com/2020/03/20/world-order-after-coroanvirus-pandemic/.

